# From Listening to Lasting Change: A Quality Improvement Initiative to Improve CRNA and CAA Retention and Recruitment

**DOI:** 10.21203/rs.3.rs-11145474/v1

**Published:** 2026-11-01

**Authors:** Savita Sharma, Colby Simmons, Tobias de la Garza Eckle

## Abstract

**Background:** The COVID-19 pandemic created unprecedented workforce challenges within anesthesia departments across the United States. High turnover, burnout, and staffing shortages threatened patient access, operational efficiency, and financial performance. **Methods:** We conducted one-on-one, in-person interviews with 85 Advanced Practice Providers (APPs) to identify core issues driving resignation. Interviews identified low incentives, unreliable breaks, rigid scheduling, limited team-based care, and a toxic work environment as key contributors. In response, we implemented a comprehensive retention initiative to strengthen culture and improve operational efficiency. As a control, we aligned our data against the American Society of Anesthesiologists (ASA) and Somnia Workforce nationwide benchmarks. **Results:** Post-implementation, the department successfully decoupled from the collapsing national trends. While the broader U.S. anesthesia market remained unstable, our average annual voluntary resignations decreased by 53% (from 7.0 to 3.25 per year), culminating in zero voluntary resignations in 2026. Concurrently, annual recruitment volume expanded by 14.4% (increasing from 11.8 to 13.5 hires), generating a net positive variance of +41 staff over 4 years and completely reversing the 2022 staffing deficit (p = 0.035, Wilcoxon matched-pairs signed-rank test). The post-implementation survey revealed a 92% provider satisfaction rate, compared to persistent nationwide retention challenges. Economically, the initiative generated $4,862,840 in net financial profit, a 243.1% Return on Investment (ROI), a 5-year adjusted Net Present Value (NPV) of $5,038,139, and a Modified Internal Rate of Return (MIRR) of 35.84%. **Conclusion:** One-on-one interviews with APPs are a highly effective, practical, and rapid tool for identifying specific and granular drivers of attrition. Addressing these granular and group-specific issues can significantly improve retention, boost engagement, reverse a staffing crisis, and yield substantial capital preservation and institutional wealth generation.

